# Diagnostic Performances of Different Genome Amplification Assays for the Detection of Swine Vesicular Disease Virus in Relation to Genomic Lineages That Circulated in Italy

**DOI:** 10.3390/v12111336

**Published:** 2020-11-20

**Authors:** Giulia Pezzoni, Dennis Benedetti, Arianna Bregoli, Ilaria Barbieri, Efrem Alessandro Foglia, Santina Grazioli, Emiliana Brocchi

**Affiliations:** National/OIE/FAO Reference Centre for Foot-and-Mouth Disease and for Swine Vesicular Disease, Istituto Zooprofilattico Sperimentale della Lombardia e dell’Emilia Romagna (IZSLER), via Bianchi 9, 24125 Brescia, Italy; benedetti.dennis@gmail.com (D.B.); arianna.bregoli@izsler.it (A.B.); ilaria.barbieri@izsler.it (I.B.); e.foglia@izsler.it (E.A.F.); santina.grazioli@izsler.it (S.G.)

**Keywords:** SVDV, genome amplification assays, genomic lineages, field fecal samples

## Abstract

During the last 25 years, swine vesicular disease (SVD) has occurred in Italy mostly sub-clinically. Therefore, regular testing of fecal samples from suspected holdings and high turnover premises was fundamental to identifying virus circulation and to achieve SVD eradication. In this study, we evaluated diagnostic performances of six genomic amplification methods, using positive fecal samples from 78 different outbreaks (1997–2014), which included different lineages. Comparison of three RT-PCRs, designed to amplify the same 154 nt portion of the gene 3D, demonstrated that a conventional and a real-time based on SYBR Green detection assay showed the highest diagnostic sensitivity, detecting all samples, while a real-time TaqMan-based test missed three cases, owing to two mismatches in the probe target sequence. Diagnostic and analytical specificities were optimal, as 300 negative field samples and other enteroviruses reacted negative. Three further evaluated tests, previously described, were a 3D-targeted reverse transcriptase loop-mediated isothermal amplification (RT-LAMP) and two real-time RT-PCRs targeted on the 5′UTR region. Here, the presence of multiple mismatches in probe and primers reduced the diagnostic performances, and two of the assays were unable to detect viruses from one sub-lineage. These results highlight that the choice of tests using less nucleotide targets significantly contributed to the success of the SVD eradication plan.

## 1. Introduction

Swine vesicular disease virus (SVDV) belongs to the genus Enterovirus within the *Picornaviridae* family [1]. It is a non-enveloped virus with a positive single-stranded RNA genome of about 7.4 kb; its genome is organized in one open reading frame (ORF) flanked at the 5′ and 3′ ends by untranslated regions (UTRs). This unique ORF encodes for four structural proteins (VP1, VP2, VP3, and VP4) and seven non-structural proteins (2A, 2B, 2C, 3A, 3B, 3C, and 3D). SVDV is antigenically and genetically related to the human enterovirus, coxsackievirus B5 (CV-B5) [2], from which it has likely evolved [3,4].

Swine vesicular disease (SVD) is clinically characterized by the formation of erosions and vesicles on the hooves and around the mouth. The clinical signs can vary in severity, with recent outbreaks reporting a shift toward subclinical disease in infected pigs [5]. When the disease occurs clinically, the vesicular lesions are morphologically indistinguishable from those produced in the other vesicular conditions affecting pigs, such as those caused by foot-and-mouth disease virus (FMDV), vesicular stomatitis virus (VSV), vesicular exanthema of swine virus (VESV), and Seneca Valley virus A (SVV).

Both clinically and subclinically SVDV infected pigs shed the virus in feces, usually up to one month after infection, contaminating the surrounding environment [6]. Therefore, SVDV infection can be acquired through direct contact with infected pigs or exposure to a contaminated environment [7]. Movement of subclinically infected animals is considered the most common means of spreading SVDV [5,8].

SVD was first identified in Italy in 1966 [9]. Later on, epidemics of SVD occurred in some European countries and Eastern Asia during the 1970s and early 1980s. The disease continued to persist in Italy and reappeared in the European Union, outside Italy, on sporadic occasions since 1992. Although SVDV occurs as a single enterovirus serotype, four congruent genetic and antigenic clusters have been identified [1,10]: the first group consists solely of the earliest SVDV isolate (ITL1/66); the second group comprises viruses that circulated in Europe and the Far East during the 1970s and 1980s; the third includes viruses isolated from 1988–1992 only in Italy; and the fourth variant was first reported in The Netherlands and Italy (1992) and then sporadically also in Spain and Portugal. The fourth antigenic variant persisted only in Italy for more than 20 years until the last outbreak was recorded in 2015. Phylogenetic analysis on the VP1 coding region, conducted by Knowles et al. in 2007 [11], showed that the Italian SVDV isolates of the fourth antigenic group cluster in two distinguishable sub-lineages. The same study revealed that some viruses from outbreaks in central and southern Italy from 2004 were closely related to the viruses detected in Portugal in 2003/2004/2007.

Starting in 1995, Italy has implemented a national surveillance and eradication program for SVD, aimed at achieving eradication through an SVD health certification scheme for pig farms. Details of the program, which is revised annually according to the epidemiological situation, have been described elsewhere [5,8]. Owing to the sub-clinical course of SVD, active serological and virological surveillance, based on laboratory testing, were fundamental for effective disease control and eradication, which was formally recognized by the European Union in 2019 [12]. Despite achievement of eradication, the SVD surveillance program is still in place in Italy.

The SVD diagnostic procedures adopted for the implementation of the national program are those introduced at the community level in Commission Decision 2000/428/EC (https://eur-lex.europa.eu/legal-content/EN/TXT/HTML/?uri=CELEX:32000D0428&rid=6). An essential part of the program to monitor virus circulation includes testing of fecal samples for virus detection. Accordingly, a monthly virological check is carried out in dealers’ premises and in fattening farms characterized by high turnover operations [12]. For SVDV detection in feces, the virus isolation test was used up to 2002, and then replaced with the more performant reverse transcriptase–polymerase chain reaction (RT-PCR) [5,13]. This conventional one-step RT-PCR, based on 3D coding gene, was routinely used in our National Reference Laboratory for many years. However, the availability of a screening test for virus detection as performant as the conventional RT-PCR from the diagnostic point of view, but with the advantages of a real-time RT-PCR procedure or the easiness of an RT-LAMP, was strongly desirable.

A variety of RT-PCR methods have been described for the detection of SVDV, based on both conventional [14,15,16] and real-time (rt) formats [17,18,19]; some of these assays are designed in a multiplex format to differentiate SVDV from FMDV and VSV [20,21]. A one-step reverse transcriptase loop-mediated isothermal amplification (RT-LAMP) assay has also been developed [22]. As the RT-LAMP can be performed without using a thermocycler, and the results can be easily visualized [23,24], it can be transferred to simply equipped laboratories or onto a lateral flow device to be used in the field [25]. However, none of these genome amplification assays has been validated using field samples representative of the SVDV different lineages, and particularly, on matrices such as fecal samples, which are more problematic and contain significantly lower amounts of the virus compared to the vesicular tissues.

Therefore, this study aimed to develop sensitive molecular assays capable of detecting all SVDV sub-lineages and to compare the diagnostic performances of the newly developed amplification methods with those previously described, using the wide panel of SVDV positive and negative field samples from our collection. In particular, we compared the conventional one-step RT-PCR based on 3D coding gene with two newly developed real-time RT-PCRs targeted on the same genomic region. The comparison was further extended to three molecular tests, previously described, namely two real-time RT-PCRs designed on the 5′UTR region [17] and the RT-LAMP targeted on 3D [22]. The diagnostic performances of the different genomic amplification assays were then evaluated in relation to the genomic lineages that have circulated in Italy from 1997 to 2014.

## 2. Materials and Methods

### 2.1. Samples and Viruses

Fecal samples, collected from 78 different SVD outbreaks occurred in Italy during the period 1997–2014, were used for the evaluation of the diagnostic sensitivity of the genomic amplification assays. One sample from each outbreak was included. The ascertained positivity to SVDV of the 78 archived samples was based on criteria outlined in the Commission Decision 200/428/EC; these include either isolation of SVDV in cell cultures or a positive result in RT-PCR combined with another evidence of infection, such as the detection of specific antibodies in the herd of origin or an epidemiological correlation with a positive farm. Three hundred field fecal samples originating from SVDV-free Italian regions were used for the evaluation of diagnostic specificity. SVDV isolates R1394 (Firenze, Tuscany, 2002) and R1567 (Caserta, Campania, 2007) were used as reference strains representative of the two sub-lineages identified in the antigenic/genomic group 4: sub-lineage 1 and sub-lineage 2, respectively; SVDV UKG 27/72 (GenBank accession no. X54521.1) was selected as an international reference strain (second antigenic group). The reference SVDV isolates were propagated in IBRS2 cell monolayers [26] and titrated by the calculation of TCID50/mL [27]. Seven porcine teschoviruses (PTV) and three porcine enteric viruses (PEV) were used as cell-free supernatants, derived from passages of viruses in IBRS2 cells, to assess analytical specificity. Details of all the field samples and the reference viral strains used in this study are provided in Table S1.

### 2.2. RNA Extraction

In order eliminate PCR inhibitors that can be present in feces, an SVDV immune-purification step, mediated by the monoclonal antibody 5B7, was carried out before RNA extraction [13]. The detailed protocol is described in the Manual of Diagnostic Tests and Vaccines for Terrestrial Animals from the World Organisation for Animal Health (https://www.oie.int/fileadmin/Home/eng/Health_standards/tahm/3.08.08_SVD.pdf). Briefly, the fecal suspension was distributed into three wells of a 5B7-coated 96-well microplate (200 µL/well, 600 µL of the sample in total). After incubation for 1 h at 37 °C and a washing step with PBS, RNA was extracted from each sample by adding approximately 100 μL/well of lysis buffer (4 M guanidine thiocyanate, 25 mM sodium citrate, pH 7, 0.5% Sarkosyl); then, the extracted RNA (in 300 μL from three wells) was precipitated by adding a mixture of ice-cold ethanol (2 volumes), sodium acetate 3 M (pH 5.2) (0.1 volume), and glycogen (Merck Life Science, Milano, Italy). After centrifugation, the pellet was washed with ice-cold 70% ethanol, and after centrifugation, the RNA was resuspended in 20 µL of RNase free water.

The viral RNAs of PV and PEV (Table S1) were extracted from 250 µL of supernatants of infected IB-RS-2 cell cultures using Trizol reagent (Invitrogen, Carlsbad, CA, USA), according to the manufacturer’s instructions, and resuspended in 20 µL of RNase free water.

### 2.3. Genomic Sequencing and Phylogenetic Analysis

Viral RNAs extracted from the fecal samples were amplified using the primer combination reverse P2 (5′-ATATTCGTGCTCCCCGTTGTGC-3′) and forward P1 (5′-CCTCTGTAATTCCCACCACCTGTA-3′). The target region of these primers is a fragment of 487 bp within the 3D gene, corresponding to nucleotides 6715–7201 of the reference isolate UKG 27/72.

A portion of approximately 579 bp of the 5′UTR region of 26 samples has also been sequenced using two overlapping amplicons of 389 and 342 bp encompassing the target regions of the two real-time RT-PCR assays: 2B-IR and 3-IR, respectively. The following primers were used: FWD ITL 1/92 (5′-TACCTTTGTGCGCCTGTTTGACC-3′) and REV ITL 1/92 (5′-GCCGGAGGACTACCAACTAGC-3′) for the 2B-IR region; FWD ITL 19/92 (5′-TGGGACAACTCATGGGACGCTAC-3′) and REV ITL 19/92 (5′-GACGGTGTTGAGGGGCAGTTA-3′) for the 3-IR region, designed, respectively, on the SVDV isolates named ITL 1/92 and ITL 19/92 (GenBank accession number AY875998.1 and AY875995.1).

For sequencing, the amplified products were separated into an agarose gel and then purified with the Qiaquick gel extraction kit (Qiagen, Hilden, Germany). The purified PCR products were sequenced on both strands using the BigDye Terminator Cycle Sequencing kit v. 1.1 (Applied Biosystems, Carlsbad, USA) and employing the same forward and reverse primers used for the amplification. The sequence reactions were run on an ABI 3130 DNA automatic sequencer (Applied Biosystems). The sequences obtained were assembled and edited using the Lasergene Sequencing analysis software package (DNASTAR, Madison, USA), excluding the primers’ regions. A neighbor-joining phylogenetic tree using Kimura 2-parameter model with 1000 bootstrap replications was then created using the upon multiple sequence alignments by ClustalW using MEGA version 6 [28].

### 2.4. Genome Amplification Assays

The six genome amplification assays evaluated in the present study are shown in Table 1. Appropriate negative controls (RNA extracted from non-infected feces or RNase free water) and a calibrated weak positive control were included in all the amplification assays to monitor PCR accuracy.

#### 2.4.1. Conventional One-Step RT-PCR

The conventional one-step RT-PCR was carried out as described in the Manual of Diagnostic Tests and Vaccines for Terrestrial Animals from the World Organisation for Animal Health (https://www.oie.int/fileadmin/Home/eng/Health_standards/tahm/3.08.08_SVD.pdf). The reverse transcription and PCR were performed in the same tube using the Qiagen One-step RT-PCR kit according to the manufacturer’s instructions. The amplified region of 154 bp corresponds to nucleotides 6875–7028 of the reference isolate UKG 27/72 within the SVDV RNA polymerase 3D gene. The amplicon was verified through electrophoresis using a 2% agarose gel stained with SYBR Green (Euroclone, Milano, Italy).

#### 2.4.2. One-Step SYBR Green rtRT-PCR

This assay consists of an adaptation of the conventional RT-PCR, described above, in a real-time format using SYBR Green as the detector. The primers used are the same as those of the conventional RT-PCR. The reverse transcription and PCR were performed in the same tube using the QuantiTect SYBR Green RT-PCR Kit (Qiagen, Hilden, Germany) according to the manufacturer’s instructions with 0.5 µM of each primer. The amplification program used was 30 min at 50 °C (RT), 15 min at 95 °C, followed by 40 cycles consisting of 15 s at 94 °C, 30 s at 58 °C, 30 s at 72 °C, and 77 °C for 15 s with fluorescence detection. After the amplification, a melting analysis was conducted with the following profile: 1 min at 72 °C, followed by a temperature-increasing step ranging from 72–95 °C with increments of 0.5 °C for 5 s during which the fluorescence was detected.

#### 2.4.3. One-Step rtRT-PCR Using TaqMan Probe 3D Target

This assay consists of an adaptation of the conventional RT-PCR, described above, in a real-time format using a TaqMan probe. The primers used are the same as those of the conventional RT-PCR. Two different probes were mixed, of which one was for the detection of the strains belonging to sub-lineage 1 and the other for the detection of sub-lineage 2: HEX-5′ATGTGATAGCTTCATACCCGTGGC3′-BHQ and HEX-5′ATGTAATAGCTTCGTACCCGTGGC3′-BHQ. They were designed by the alignment of the portion of the 3D coding regions used for the phylogenesis of SVDV strains. The reverse transcription and PCR were performed in the same tube using the SuperScript III Platinum One-Step qRT-PCR Kit (Invitrogen, Carlsbad, USA) according to the manufacturer’s instructions, with 1 µM of each probe and 0.3 µM and 0.9 µM of the forward and reverse primers, respectively. The amplification program used was 30 min at 50 °C (RT), 15 min at 95 °C, followed by 40 cycles consisting of 20 s at 94 °C and 20 s at 58 °C.

#### 2.4.4. One-Step RT-LAMP

The RT-LAMP reaction was performed as previously described [22] using six different primers. They were designed to recognize eight distinct regions of the target sequence (214 bp) corresponding to nucleotides 6870–7083 of the reference isolate UKG 27/72 within the 3D gene. The products were visualized using electrophoresis on a 1.5% agarose gel stained with SYBR Green (Euroclone, Milano, Italy).

#### 2.4.5. One-Step rtRT-PCR Using the TaqMan Probe 5′UTR Target

The two rtRT-PCR reactions having the 5′UTR region as a target were performed as previously described by Reid et al., 2004 [17]; the assays involved two different portions within the 5′UTR region, named 3-IR and 2B-IR. The reverse transcription and PCR were performed in the same tube using the SuperScript III Platinum One-Step qRT-PCR Kit (Invitrogen, Carlsbad, USA) according to the manufacturer’s instructions.

## 3. Results

### 3.1. Analytical Sensitivity and Specificity of the Genome Amplification Assays

The analytical sensitivity of the six genomic amplification assays (Table 1) was evaluated using three SVDV isolates, grown in cell cultures and titrated, R1394 (Italy 2002) and R1567 (Italy 2007) representing the previously identified sub-lineages 1 and 2, respectively, within the fourth antigenic/genomic group [10,11] and UKG 27/72 representing the second antigenic/genomic group. Tenfold serial dilutions of each isolate were performed in SVDV negative fecal samples, the RNA was extracted and amplified in triplicates using the six molecular assays.

The two real-time RT-PCRs targeted on the 3D region (based on SYBR Green and TaqMan probe detection) and the 2B-IR rtRT-PCR targeted on the 5′UTR region showed an equivalent analytical sensitivity, corresponding to the detection limit of 10 TCID50. The conventional one-step RT-PCR (3D) and the real-time assay named 3-IR (5′UTR) showed slightly higher sensitivity, being able to detect 1 TCID50 (Table 2); however, interestingly, the latter test was not able to detect the strain R1567, representative of sub-lineage 2, even at the highest virus doses. The less sensitive test resulted the one-step RT-LAMP, which showed a detection limit of 10^2^ TCID50 when the SVDV isolates R1394 and UKG 27/72 were examined, whilst a massive viral payload of 10^6.6^ TCID50 was necessary to obtain a positive amplification signal when the SVDV isolate R1567 (sub-lineage 2) was examined.

Analytical specificity was evaluated for the three RT-PCRs targeted on 3D, namely the conventional assay and the two real-time tests, with evidence of 100% specificity. It was not evaluated for the other three previously described tests.

### 3.2. Diagnostic Sensitivity and Specificity of the Genome Amplification Assays

The evaluation of diagnostic sensitivity was undertaken on the field fecal samples that originated from 78 different SVDV outbreaks collected during the period 1997–2014. The conventional one-step RT-PCR as well as the SYBR Green rtRT-PCR, both using the same primers on the 3D region, detected 100% of the strains of either genomic sub-lineages (Table 3). In the latter test, the melting temperature of the specific amplificon resulted in the range of 79.9–82 °C.

The 3D-based rtRT-PCR with the TaqMan probe missed three strains belonging to sub-lineage 1 (Table 3). Of the two real-time tests performed on the 5′UTR region, the 2B-IR rtRT-PCR missed six strains belonging to sub-lineage 1, while detecting all samples of sub-lineage 2. In contrast, the 3-IR rtRT-PCR did not detect any of the strains belonging to sub-lineage 2 and further 12 samples containing viruses belonging to sub-lineage 1 (Table 3). The RT-LAMP test performed poorly: it missed all the sub-lineage 2 strains similarly to the 3-IR rtRT-PCR, in addition to 14 from sub-lineage 1 (Table 3).

Diagnostic specificity was evaluated for the three RT-PCRs targeted on 3D: all the 300 field samples that originated from SVDV-free Italian regions scored negative, resulting in 100% specificity.

### 3.3. Sequencing of Genomic Regions Target of the Amplification Tests and Phylogenetic Analysis of the SVDV Circulated in Italy

To get more insight into the different performances of the genomic amplification assays evaluated in the present study, the nucleotide sequences of portions of the 3D (433 bp) and 5′UTR (573–579 bp) regions, including the target of the assays, were obtained from the samples. The alignment of 3D sequences showed the presence of several mismatches within the RT-LAMP target regions of all the undetected strains, as shown in Figure S1. The number and distribution of mismatches was different among the viruses of the two sub-lineages, and even among strains of the same sub-lineage; some of the mismatches were commonly present in both detected and undetected samples.

In two out of three undetected strains using the 3D rtRT-PCR, two mismatches were found in the TaqMan probe region of which one was in common with other detected strains.

**Figure 1:**
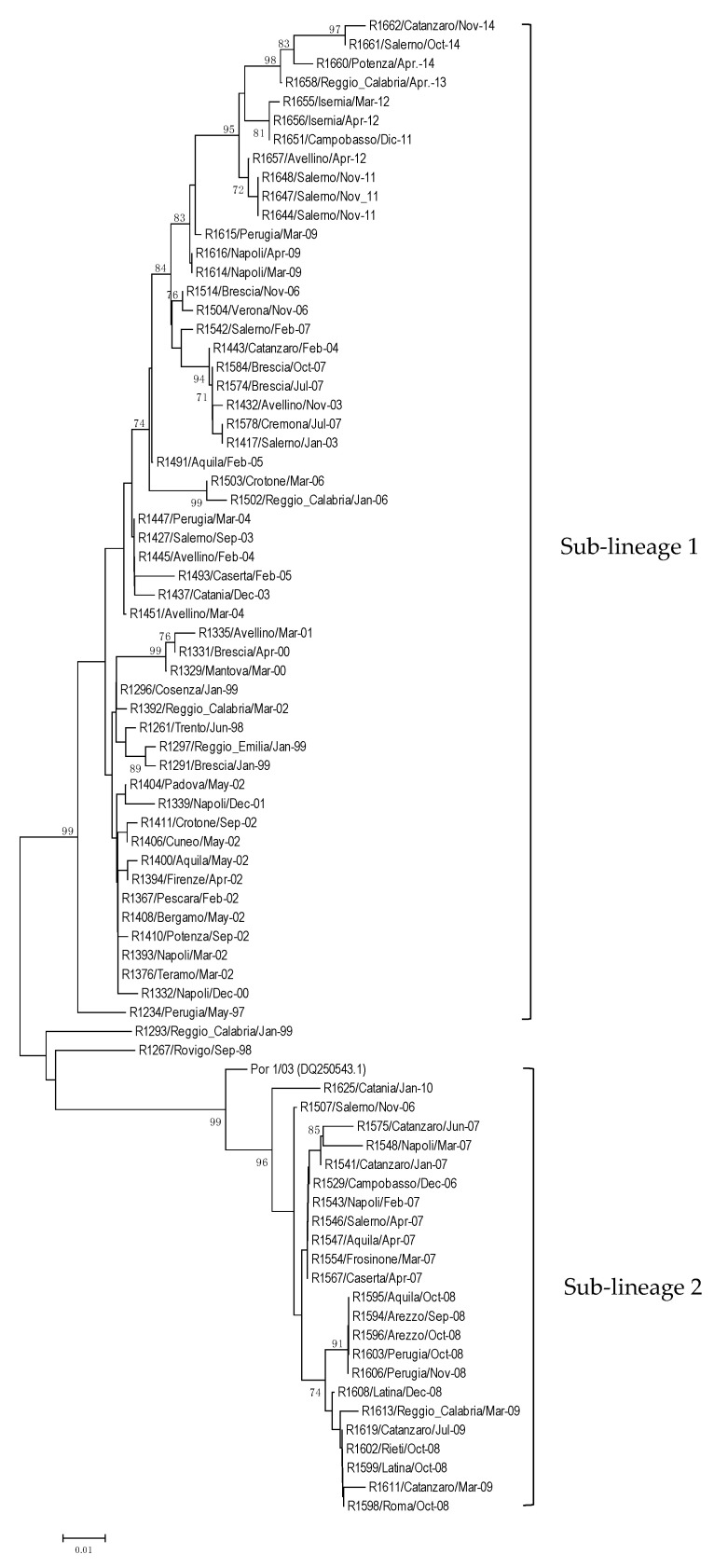
Phylogenetic tree derived from a nucleotide sequence fragment (433 nt) of 3D gene from the 78 Italian SVDV-positive samples (the Portuguese strain Por 1/03 retrieved from GenBank is also included). The two viral clusters named sub-lineage 1 and 2 correspond to those identified by the phylogenesis based on VP1 sequences. The designation of tree reconstruction is performed as described in the materials and methods using the program MEGA neighbor-joining method and bootstrapped with 1000 replicates. Only bootstrap values higher than 70% are shown.

According to the constructed neighbor-joining tree, the 78 SVDV positive samples examined could be grouped into two genomic sub-lineages (Figure 1), consistent with previous observations based on the VP1 gene [11]. One cluster, referred to as “sub-lineage 1,” comprises 55 viruses from outbreaks that occurred during the period under consideration (1997–2014) and were distributed throughout the country (99% bootstrap support). The other, referred to as “sub-lineage 2,” includes viruses detected during 2006–2010 from the central/southern regions of Italy (99% bootstrap support), which are closely related to viral strains isolated in Portugal in 2003/2004 (Figure 1). According to Knowles et al. [11], this lineage of viruses also includes the Portuguese isolates from 2007.

When the 3D partial nucleotide sequences of both sub-lineages were compared to that of the SVDV strain R1394, selected as representative for sub-lineage 1, the strains belonging to sub-lineages 1 and 2 showed a sequence identity ranging from 93–99.5% and 89.1–90.7%, respectively.

A selection of 26 samples from both sub-lineages (19 for lineage 1 and seven for lineage 2) was also sequenced at the 5′UTR region. Up to three simultaneous mismatches, causing failure of the 2B-IR rtRT-PCR, were identified within the forward primer, while the presence of two simultaneous mismatches within the probe of the 3-IR rtRT-PCR was responsible for the failure of this amplification test (Figures S2 and S3).

In contrast to the classification within sub-lineage 1 based on the 3D sequence, 12 samples showed a 5′UTR sequence identities higher with strains belonging to sub-lineage 2 (90–98.8%) than with sub-lineage 1 strains (86.2–91%).

## 4. Discussion

In Italy, SVD has been present from 1966 to 2015. The SVD-free status was achieved through the implementation of a national surveillance and eradication plan based on active serological and virological investigations.

In this study, we evaluated the diagnostic performances of six molecular diagnostic techniques in relation to genomic lineages that have circulated in Italy. The evaluation was made possible thanks to the unique and largest collection of SVDV positive samples, collected and conserved during surveillance activities conducted in compliance with the national eradication plan. Given the absence of clinical signs, the samples of choice for virus detection to identify sub-clinical infections were feces.

We analyzed field fecal samples originating from 78 outbreaks that occurred in Italy from 1997–2014. The study included sequencing of portions of 3D and 5′UTR, within which are located the target regions for the six assays under evaluation. A phylogenetic analysis conducted on a portion of about 433 nt of the 3D gene confirmed the presence of two different lineages in Italy, consistent with previous results obtained using the VP1 coding region [11]. Sub-lineage 1 includes SVD viruses that evolved in Italy and were identified in outbreaks recorded in central/southern Italy, with sporadic spillover in the northern regions; sub-lineage 2 includes viruses that circulated from 2004, related to those detected in Portugal between 2003 and 2007.

The comparison of the six genomic detection techniques showed differences in their capability to detect strains of the two SVDV genomic sub-lineages. The one-step conventional RT-PCR and the rtRT-PCR with SYBR Green detection, both using the same primer pair to amplify the same 3D fragment, recognized all the positive samples; the other four assays missed some samples belonging to sub-lineage 1, and in addition, two of them (3-IR rtRT-PCR and RT-LAMP) did not detect any of the samples containing strains belonging to sub-lineage 2 (Table 3). The alignment of the 3D and 5′UTR sequences highlighted nt mismatches in the target regions of primers or probes for the missed samples, which could explain the failure of detection. Table 4 provides an overview of such mismatches affecting the different biomolecular assays, while the supplementary figures show details of positions and numbers of the nt mismatches in the undetected samples.

In particular, for the 3D rtRT-PCR, the occurrence of a second mismatch within the TaqMan probe region of two undetected strains caused the lack of reaction, even with high viral load (data not showed), while for the third undetected sample showing a single mismatch, the reaction failure was more likely attributable to an insufficient quantity of the virus. For the RT-LAMP, several mismatches within the primers sequences were detected; however, the missed detection of 14 SVDV samples of sub-lineage 1 (25%) was more likely owed to the lower analytical sensitivity of this test combined with a low amount of SVDV in these fecal samples rather than to specific mismatches. In fact, some nucleotide mismatches were identified in the target sequences of the primers of all the 55 strains of sub-lineage 1, without correlation with a positive or negative outcome of the test. The strains belonging to sub-lineage 2, all undetectable by the RT-LAMP test, showed six common mutations within the target sequences of the primers, which were not present in strains of sub-lineage 1 (in addition to few further mismatches differently distributed); these specific nt substitutions are supposed to adversely affect the reaction (Figure S1).

These findings are consistent with the results of analytical sensitivity studies. Using the SVDV strain R1394 as a representative for sub-lineage 1, we found that the RT-LAMP detection limit was 10^2^ TCID50, while all the other genomic assays were able to detect at least 10 TCID50. The difference in analytical sensitivity appeared amplified when the SVDV strain R1567 was considered as a representative for sub-lineage 2. In this case, the RT-LAMP reaction was at least 10^5.6^-fold less sensitive than the other assays. Thus, the presence of mutations in the target sequence of the assay affects the sensitivity performance of the reaction in conjunction with the amount of the analyte. Concerning the two real-time amplification assays targeted in the 5′UTR region, the sequencing highlighted the presence of mismatching within the primers or probe regions. In particular, from 1–3 mismatches were found close to the 3′ end of the forward primer and one within the reverse primer for the six samples of sub-lineage 1 undetected by the 2B-IR real-time assay (Figure S2). After the degeneration of some of the mismatched bases, three out of six samples became detectable, and the other three were detected if a high viral payload—obtained by virus propagation in cell cultures—was tested (data not showed). Thus, the mismatches at the primers level reduce the efficiency of amplification, affecting the analytical sensitivity of the reaction. Regarding the real-time RT-PCR named 3-IR, the comparison of the 5′UTR sequences highlighted the mismatch of the same two bases at the probe target sequence of all the undetected samples that have been sequenced (12 strains from sub-lineage 1 and seven strains belonging to sub-lineage 2), while the presence of only a single mismatch was tolerated (Figure S3). Interestingly, the sequence of the 5′UTR region of the 12 undetected strains that were based on the 3D coding region were classified in sub-lineage 1 and showed a higher range of similarity with strains of sub-lineage 2 (90–98.8%) than with those of the homologous sub-lineage 1 (86.2–91%); indeed, they clustered within sub-lineage 2 in a phylogenetic tree constructed using 5′UTR sequences (not shown). These data, which are inconsistent with those obtained for the phylogenetic analysis based on the 3D coding gene, suggest that a recombination event may have occurred between strains of the two sub-lineages. Further insight into the SVD virus evolution, investigated by comparison of the full genome sequences, will be presented in a subsequent study.

Overall, the most sensitive and performant assays for SVDV detection in fecal samples were the conventional 3D RT-PCR and the 3D rtRT-PCR using SYBR Green as a detector, which both use the same primers to amplify the same region of the genome. In addition, the 3D rtRT-PCR using the TaqMan probe and based on the same primers showed an acceptable diagnostic sensitivity, though three samples remained undetected. These three assays showed 100% analytical and diagnostic specificity based on the parallel testing of other porcine enteroviruses and 300 negative field fecal samples of known origin. In addition, it is noteworthy that the presence of enteroviruses other than SVDV in at least 25% of the field fecal samples examined during routine testing (more than 2500 samples/year, personal communication) did not cause any interference in the two 3D amplification assays (conventional and real-time with SYBR Green detection) that have been sequentially adopted for the national eradication program.

Regarding the other techniques, all were affected by multiple mismatching at the probe or primers level, which strongly reduced the diagnostic performance. Owing to the subclinical nature of SVD, as observed in most outbreaks reported in recent years, laboratory confirmation of the diagnosis must rely on the testing of fecal samples, which are characterized by low virus content and, therefore, require sensitive assays. Furthermore, the unexpected high nt mutation rate observed in the genomic regions selected as target for PCR (5′UTR and 3D) highlighted the difficulty in identifying a molecular amplification test capable of detecting all virus lineages with a homogeneous efficiency. This explains why the rtRT-PCR with SYBR Green detection and the conventional RT-PCR, both using only two primers within the 3D region, have less chance of incurring nucleotide mutations and thus provide the best diagnostic sensitivity. These are the tests of choice adopted for routine virological surveillance in Italy, performed as laid down in the national program. The conventional one-step RT-PCR has been routinely used in the Italian National Reference Laboratory for vesicular diseases for almost 20 years, from 2002, when it replaced the virus isolation test, until 2018 when the SYBR Green rtRT-PCR was introduced. Through an efficient and sensitive detection of all outbreaks and circulating viruses, these assays contributed to the success in disease eradication.

## Figures and Tables

**Table 1:** Molecular assays evaluated in the study.

SVDV Biomolecular Methods	SVDV Genome Target	Nt Region According to UKG 27/72 (X54521.1)	Reference
Conventional RT-PCR	3D	6875–7028 nt	[13]
rtRT-PCR with SYBR Green	3D	6875–7028 nt	This study
rtRT-PCR with TaqMan probe	3D	6875–7028 nt	This study
LAMP RT-PCR	3D	6870–7083 nt	[22]
rtRT-PCR 2B-IR with TaqMan probe	5′UTR	252–332 nt	[17]
rtRT-PCR 3-IR with TaqMan probe	5′UTR	455–522 nt	[17]

**Table 2:** Comparison of the analytical sensitivity of the six molecular assays.

Strain	3D-Based Amplification Reactions				5′UTR Based rtRT-PCR	
	RT-PCR Conventional	rtRT-PCR SYBR Green	rtRT-PCR TaqMan Probe	RT-LAMP	2B-IR TaqMan Probe	3-IR TaqMan Probe
R1394	1	10	10	10^2^	10	1
UKG 27/72	1	10	10	10^2^	10	1
R1567	1	10	10	10^6^.^6^	10	Not detected

Values indicate the detection limits expressed as TCID50, average of three replicates, observed for three reference strains: R1394 representative for the genomic sub-lineage 1, R1567 representative for the genomic sub-lineage 2, UKG 27/72 historical reference virus representative for the second antigenic group.

**Table 3:** Comparative diagnostic sensitivity of the six molecular assays tested on 78 fecal samples, each collected from different SVDV outbreaks that occurred in Italy from 1997–2014.

SVDV Lineage		3D-Based Amplification Reactions				5′UTR rtRT-PCR	
		RT-PCR Conventional	rtRT-PCR SYBR Green	rtRT-PCR TaqMan	RT-LAMP	2B-IR TaqMan	3-IR TaqMan
Sublineage-1 (55 samples)	Assays concordance	33	33	33	33	33	33
		6	6	6	0	6	6
		4	4	4	4	0	4
		4	4	4	4	4	0
		5	5	5	0	5	0
		1	1	0	0	1	0
		2	2	0	0	0	0
	Total positive samples	55	55	52	41	49	43
		100%	100%	95%	75%	89%	78%
Sublineage-2 (23 samples)	Assays concordance	23	23	23	0	23	0
	Total positive samples	23	23	23	0	23	0
		100%	100%	100%	0%	100%	0%

**Table 4:** Evidence of nt mismatches within primers/probe sequences, causing different levels of failure of the SVDV biomolecular assays.

Target Region	SVDV Molecular Assay	Ratio Undetected/ Total Samples	Presence of Mismatches at Primers/Probe Level and Probable Cause of Reaction Failure
3D	RT-PCR conventional	None	None or few tolerated mismatches within primers
	rtRT-PCR SYBR Green	None	None or few tolerated mismatches within primers
	rtRT-PCR TaqMan	3/78 (sub-lin 1)	n. 2 mismatches within probe = reaction failure (n. 1 mismatch within probe = tolerated)
	RT-LAMP	14/55 sub-lin 1	Several mismatches within primers, irrespective reaction output = reaction failure due to lower analytical sensitivity
		23/23 sub-lin 2	Additional six common mismatches = reaction failure
5′UTR	rt2B-IR TaqMan	6/78 (sub-lin 2)	1–3 specific mismatches within primer forward = reaction failure for six samples
5′UTR	rt3-IR TaqMan	12/55 sub-lin 1	n. 2 mismatches within probe = reaction failure (n. 1 mismatch within probe = tolerated)
		23/23 sub-lin 2	

## Supplementary Materials

The following are available online at https://www.mdpi.com/1999-4915/12/11/1336/s1: Table S1: Designation and origin of SVDV, porcine teschoviruses (PTV), and porcine enteroviruses (PEV) studied. Figure S1. Alignment of SVDV-3D sequences (including the nucleotide region 6870–7083 according to the reference strain UKG 27/72 (X54521.1)) showing mismatches for the 14 samples belonging to the sub-lineage 1 undetected in RT-LAMP. Figure S2. Alignment of SVDV-5′UTR sequences (including the nucleotide region 252–332 according to the reference strain UKG 27/72 (X54521.1)) showing mismatches for the six samples belonging to sub-lineage 1 undetected in 2B-IR rtRT-PCR. Figure S3. Alignment of SVDV-5′UTR sequences (including nucleotide region 455–522 according to the reference strain UKG 27/72 (X54521.1)) showing mismatches found in a representative selection of seven samples belonging to sub-lineage 1 and two samples belonging to sub-lineage 2 (*) undetected in the 3-IR real-time RT-PCR.
