# Supplementary material for: Diagnostic Performances of Different Genome Amplification Assays for the Detection of Swine Vesicular Disease Virus in Relation to Genomic Lineages That Circulated in Italy
Source: Viruses. 2020 Nov 20;12(11):1336. doi: 10.3390/v12111336 (PMC7699968; doi:10.3390/v12111336)
Supplement: Supplementary file 1 [file viruses-12-01336-s001.zip › Fig. S1.pdf]

Fig. S1. Alignment of SVDV-3D sequences [including the nucleotide region 6870-7083 according to the reference strain UKG 27/72 (X54521.1)] showing mismatches for the 14 samples belonging to the sub-lineage 1 undetected in RT-LAMP.

```
                          ACCAATTCAGAATGATTGCA      GATGATGTGATAGCTTCATACCCGTGGCCYATCGATGCCT  CTGCTAGCTG
                                 10        20        30        40        50        60        70        80
                        ---------+---------+---------+---------+---------+---------+---------+---------+
R1662/Catanzaro/Dec-14  .....................G....................C.....T..............C..TG............
R1661/Salerno/Oct-14    .....................G..........................T..............C..TG............
R1660/Potenza/Apr-14    ..........................................C.....T..............C..TG............
R1655/Isernia/Mar-12    ................................................T..............C...G............
R1651/Campobasso/Dic-11 ................................................T..............C...G............
R1648/Salerno/Nov-11    ................................................T..............C...G............
R1647/Salerno/Nov-11    ................................................T..............C...G............
R1644/Salerno/Nov-11    ................................................T..............C...G............
R1616/Napoli/Apr-09     ...............................................................C..TG............
R1514/Brescia/Nov-06    ...............................................................C..TG............
R1493/Caserta/Feb-05    ....................................A..........................C..TG............
R1408/Bergamo/May-02    ...............................................................C..TG............
R1332/Napoli/Dec-00     ............................................................T..C..TG............
R1329/Mantova/Mar-00    ...............................................................C..TG............
R1394/Firenze/May-02    ...............................................................C..TG............
R1567/Caserta/Apr-07    ....................................A........G....................T...........C.

                        AAGCAGGGA                        CAGCAGATAAAGGCGAGTG             ACCTGGACAAACGTG
                                 90        100       110       120       130       140       150       160
                        ---------+---------+---------+---------+---------+---------+---------+---------+
R1662/Catanzaro/Dec-14  .G.........................................G....................G...............
R1661/Salerno/Oct-14    .G.........................................G....................G...............
R1660/Potenza/Apr-14    .G..............................................................................
R1655/Isernia/Mar-12    .G..............................................................................
R1651/Campobasso/Dic-11 .G..............................................................................
R1648/Salerno/Nov-11    .G.....A........................................................................
R1647/Salerno/Nov-11    .G.....A........................................................................
R1644/Salerno/Nov-11    .G.....A........................................................................
R1616/Napoli/Apr-09     ................................................................................
R1514/Brescia/Nov-06    ................................................................................
R1493/Caserta/Feb-05    ................................................................................
R1408/Bergamo/May-02    ................................................................................
R1332/Napoli/Dec-00     ................................................................................
R1329/Mantova/Mar-00    ............................................A...................................
R1394/Firenze/May-02    ................................................................................
R1567/Caserta/Apr-07    .G...................................G......A.T........................G........

                        ACCT        AGGTAYTTTAGGGCAGAT      TACCCRTTTTTGGTCCATCC
                                 170       180       190       200       210
                        ---------+---------+---------+---------+---------+------
R1662/Catanzaro/Dec-14  .............................................C..........
R1661/Salerno/Oct-14    ....................C........................C..........
R1660/Potenza/Apr-14    ....................C........................C..........
R1655/Isernia/Mar-12    ....................C........................C..........
R1651/Campobasso/Dic-11 ....................C........................C..........
R1648/Salerno/Nov-11    ....................C........................C..........
R1647/Salerno/Nov-11    ....................C........................C..........
R1644/Salerno/Nov-11    ....................C........................C..........
R1616/Napoli/Apr-09     ..............A....C........................C..........
R1514/Brescia/Nov-06    ..............A....C........................C..........
R1493/Caserta/Feb-05    ..............A....C.....G...........T......C..........
R1408/Bergamo/May-02    ..............A....C........................C..A.......
R1332/Napoli/Dec-00     ..............A....C........................C..A.......
R1329/Mantova/Mar-00    ..............A....C........................C..A.......
R1394/Firenze/May-02    ..............A....C........................C..A.......
R1567/Caserta/Apr-07    ....................C.............................T.....
```

Footnote: Strains representative for sub-lineage 1 (R1394) and for sub-lineage 2 (R1567) are highlighted in grey. Target regions used in the RT-LAMP assay are highlighted in grey. Only nucleotide mismatches within the target regions are indicated; those found in R1567 representing sub-lineage 2 are in italic.
