# Supplementary material for: Diagnostic Performances of Different Genome Amplification Assays for the Detection of Swine Vesicular Disease Virus in Relation to Genomic Lineages That Circulated in Italy
Source: Viruses. 2020 Nov 20;12(11):1336. doi: 10.3390/v12111336 (PMC7699968; doi:10.3390/v12111336)
Supplement: Supplementary file 1 [file viruses-12-01336-s001.zip › Fig. S2.pdf]

Fig. S2. Alignment of SVDV-5’UTR sequences [including the nucleotide region 252-332 according to the reference strain UKG 27/72 (X54521.1)] showing mismatches for the six samples belonging to sub-lineage 1 undetected in 2BIR rtRT-PCR.

```
                                     CGAGAAACCTAGTACCACCATGAAA             TCGCTCCGCACAACCCCAGTG  GATCAGGTCGA
                                     10        20        30        40        50        60        70        80
                             ---------+---------+---------+---------+---------+---------+---------+---------+
R1432/Avellino/Nov-03        ............................C...............................................C...
R1502/Reggio_Calabria/Jan-06 ............................C.G.............................................C...
R1503/Crotone/Mar-06         ............................C.G.............................................C...
R1584/Brescia/Oct-06         ..........................T.C.G.............................................C...
R1660/Potenza/Apr-14         ................A.....T....G.....................T..........................C...
R1662/Catanzaro/Nov-14       ................T.......G..G.........................................A......C...
R1394/Firenze/Apr-02         ............................................................................C...
R1567/Caserta/Apr-07         ............................................................................C...

                             TGAGTCACCG

                                     90
                             ---------+
R1432/Avellino/Nov-03        ....C.....
R1502/Reggio_Calabria/Jan-06 ....C.....
R1503/Crotone/Mar-06         ....C.....
R1584/Brescia/Oct-06         ....C.....
R1660/Potenza/Apr-14         ..........
R1662/Catanzaro/Nov-14       ..........
R1394/Firenze/Apr-02         ....C.....
R1567/Caserta/Apr-07         ..........
```

Footnote: Strains representative for sub-lineage 1 (R1394) and for sub-lineage 2 (R1567) are highlighted in grey. Primers and probe regions used in the 2BIR rtRT-PCR assay are highlighted in grey and nucleotide mismatches within them are indicated; those found only in the six missed samples and thus affecting the test result are in bold italic.
