# Supplementary material for: Diagnostic Performances of Different Genome Amplification Assays for the Detection of Swine Vesicular Disease Virus in Relation to Genomic Lineages That Circulated in Italy
Source: Viruses. 2020 Nov 20;12(11):1336. doi: 10.3390/v12111336 (PMC7699968; doi:10.3390/v12111336)
Supplement: Supplementary file 1 [file viruses-12-01336-s001.zip › Fig. S3.pdf]

Fig. S3. Alignment of SVDV-5′UTR sequences [including nucleotide region 455-522 according to the reference strain UKG 27/72 (X54521.1)] showing mismatches found in a representative selection of seven samples belonging to sub-lineage 1 and two samples belonging to sub-lineage 2 (*) undetected in the 3IR Real-time RT-PCR.

```
                            CCCTGAATGCGGCTAATCC  ACTGCGGAGCAGATACCCATGCA    TGGGCAGTCTGTCGTAATGG
                                 10        20        30        40        50        60        70
                        ---------+---------+---------+---------+---------+---------+---------+---------
R1644/Salerno/Nov-11    .............................T..............T..................................
R1651/Campobasso/Dic-11 .............................T..............T..................................
R1655/Isernia/Mar-12    .............................T..............T..................................
R1660/Potenza/Apr-14    .............................T..............T..................................
R1661/Salerno/Oct-14    .............................T..............T............G.....................
R1662/Catanzaro/Nov-14  .............................T..............T............G.....................
R1554/Frosinone/Mar-07  .............................T..............T..................................
R1602/Rieti/Oct-08*     .............................T..............T..................................
R1625/Catania/Jan-10*   .............................T..............T..................................
R1394/Firenze/Ap-02     .............................T.................................................
R1567/Caserta/Ap-07     .............................T..............T..................................
```

Footnote: Strains R1394 and R1597, highlighted in grey, represent sub-lineage 1 and sub-lineage 2 respectively. Primers and probe regions used in the 3IR assay are highlighted in grey and nucleotide mismatches within them are indicated. Two nucleotide mismatches within the probe target sequence were commonly found in all the sequenced samples missed by the test (12 of sub-lineage 1 and 7 of sub-lineage 2), regardless the sub-lineage.
