# Supplementary material for: Diagnostic Performances of Different Genome Amplification Assays for the Detection of Swine Vesicular Disease Virus in Relation to Genomic Lineages That Circulated in Italy
Source: Viruses. 2020 Nov 20;12(11):1336. doi: 10.3390/v12111336 (PMC7699968; doi:10.3390/v12111336)
Supplement: Supplementary file 1 [file viruses-12-01336-s001.zip › Table S1.pdf]

TABLE S1. Designation and origin of SVDV, Porcine teschoviruses (PTV), and Porcine enteroviruses (PEV) studied.

Virus	Strain	GenBank sequence number	Sample	Geographic origin	Date of collection
SVDV	ITL/R1234/97	MT856746	Faeces	Perugia, Umbria	May 97
SVDV	ITL/R1261/98	MT856747	Faeces	Trento, Trentino Alto Adige	June 98
SVDV	ITL/R1267/98	MT856748	Faeces	Rovigo, Veneto	September 98
SVDV	ITL/R1291/99	MT856749 MT878498*	Faeces	Brescia, Lombardia	January 99
SVDV	ITL/R1293/99	MT856750	Faeces	Reggio Calabria, Calabria	January 99
SVDV	ITL/R1296/99	MT856751	Faeces	Cosenza, Calabria	January 99
SVDV	ITL/R1297/99	MT856752	Faeces	Reggio Emilia, Emilia Romagna	January 99
SVDV	ITL /R1329/00	MT856753	Faeces	Mantova, Lombardia	March 00
SVDV	ITL/R1331/00	MT856754	Faeces	Brescia, Lombardia	April 00
SVDV	ITL/R1332/00	MT856755	Faeces	Napoli, Campania	December 00
SVDV	ITL/R1335/01	MT856756	Faeces	Avellino, Campania	March 01
SVDV	ITL/R1339/01	MT856757	Faeces	Napoli, Campania	December 01
SVDV	ITL/R1367/02	MT856758	Faeces	Pescara, Abruzzo	February 02
SVDV	ITL/R1376/02	MT856759	Faeces	Teramo, Abruzzo	March 02
SVDV	ITL/R1392/02	MT856760	Faeces	Reggio Calabria, Calabria	March 02
SVDV	ITL/R1393/02	MT856761	Faeces	Napoli, Campania	March 02
SVDV	ITL/R1394/02	MT856762 MT878499*	Cc and Faeces	Firenze, Toscana	April 02
SVDV	ITL/R1400/02	MT856763	Faeces	Aquila, Abruzzo	May 02
SVDV	ITL/R1404/02	MT856764	Faeces	Padova, Veneto	May 02
SVDV	ITL/R1406/02	MT856765	Faeces	Cuneo, Piemonte	May 02
SVDV	ITL/R1408/02	MT856766	Faeces	Bergamo, Lombardia	May 02
SVDV	ITL/R1410/02	MT856767	Faeces	Potenza, Basilicata	September 02
SVDV	ITL/R1411/02	MT856768	Faeces	Crotone, Calabria	September 02
SVDV	ITL/R1417/03	MT856769	Faeces	Salerno, Campania	January 03
SVDV	ITL/R1427/03	MT856770	Faeces	Salerno, Campania	September 03
SVDV	ITL/R1432/03	MT856771 MT878500*	Faeces	Avellino, Campania	November 03
SVDV	ITL/R1437/03	MT856772	Faeces	Catania, Sicilia	December 03
SVDV	ITL/R1443/04	MT856773	Faeces	Catanzaro, Calabria	February 04
SVDV	ITL/R1445/04	MT856774	Faeces	Avellino, Campania	February 04
SVDV	ITL/R1447/04	MT856775	Faeces	Perugia, Umbria	March 04
SVDV	ITL/R1451/04	MT856776	Faeces	Avellino, Campania	March 04
SVDV	ITL/R1491/05	MT856777	Faeces	Aquila, Abruzzo	February 05
SVDV	ITL/R1493/05	MT856778	Faeces	Caserta, Campania	February 05
SVDV	ITL/R1502/05	MT856779 MT878501*	Faeces	Reggio Calabria, Calabria	January 06

SVDV	ITL/R1503/06	MT856780 MT878502*	Faeces	Crotone, Calabria	March 06
SVDV	ITL/R1504/06	MT856781	Faeces	Verona, Veneto	November 06
SVDV	ITL/R1507/06	MT856782	Faeces	Salerno, Campania	November 06
SVDV	ITL/R1514/06	MT856783	Faeces	Brescia, Lombardia	November 06
SVDV	ITL/R1529/06	MT856784	Faeces	Campobasso, Molise	December 06
SVDV	ITL/R1541/07	MT856785	Faeces	Catanzaro, Calabria	January 07
SVDV	ITL/R1542/07	MT856786	Faeces	Salerno, Campania	February 07
SVDV	ITL/R1543/07	MT856787	Faeces	Napoli, Campania	February 07
SVDV	ITL/R1546/07	MT856788	Faeces	Salerno, Campania	April 07
SVDV	ITL/R1547/07	MT856789	Faeces	Aquila, Abruzzo	April 07
SVDV	ITL/R1548/07	MT856790 MT878503*	Faeces	Napoli, Campania	March 07
SVDV	ITL/R1554/07	MT856791 MT878504*	Faeces	Frosinone, Lazio	March 07
SVDV	ITL/R1567/07	MT856792 MT878505*	Cc and Faeces	Caserta, Campania	April 07
SVDV	ITL/R1574/07	MT856793 MT878506*	Faeces	Brescia, Lombardia	July 07
SVDV	ITL/R1575/07	MT856794	Faeces	Catanzaro, Calabria	June 07
SVDV	ITL/R1578/07	MT856795	Faeces	Cremona, Lombardia	July 07
SVDV	ITL/R1584/07	MT856796 MT878507*	Faeces	Brescia, Lombardia	October 07
SVDV	ITL/R1594/08	MT856797	Faeces	Arezzo, Toscana	September 08
SVDV	ITL/R1595/08	MT856798	Faeces	Aquila, Abruzzo	October 08
SVDV	ITL/R1596/08	MT856799 MT878508*	Faeces	Arezzo, Toscana	October 08
SVDV	ITL/R1598/08	MT856800	Faeces	Roma Lazio	October 08
SVDV	ITL/R1599/08	MT856801	Faeces	Latina, Lazio	October 08
SVDV	ITL/R1602/08	MT856802 MT878509*	Faeces	Rieti, Lazio	October 08
SVDV	ITL/R1603/08	MT856803 MT878510*	Faeces	Perugia, Umbria	October 08
SVDV	ITL/R1606/08	MT856804	Faeces	Perugia, Umbria	November 08
SVDV	ITL/R1608/08	MT856805	Faeces	Latina, Lazio	December 08
SVDV	ITL/R1611/08	MT856806	Faeces	Catanzaro, Reggio Calabria	March 09
SVDV	ITL/R1613/09	MT856807	Faeces	Reggio Calabria, Calabria	March 09
SVDV	ITL/R1614/09	MT856808	Faeces	Napoli Campania	March 09
SVDV	ITL/R1615/09	MT856809 MT878511*	Faeces	Perugia, Umbria	March 09
SVDV	ITL/R1616/09	MT856810	Faeces	Napoli, Campania	April 09
SVDV	ITL/R1619/09	MT856811	Faeces	Catanzaro, Calabria	July 09
SVDV	ITL/R1625/10	MT856812 MT878512*	Faeces	Catania, Sicilia	January 10
SVDV	ITL/R1644/11	MT856813 MT878513*	Faeces	Salerno, Campania	November 11
SVDV	ITL/R1647/11	MT856814 MT878514*	Faeces	Salerno, Campania	November 11
SVDV	ITL/R1648/11	MT856815 MT878515*	Faeces	Salerno, Campania	November 11
SVDV	ITL/R1651/11	MT856816 MT878516*	Faeces	Campobasso, Basilicata	December 11

SVDV	ITL/R1655/12	MT856817 MT878517*	Faeces	Isernia, Molise	March 12
SVDV	ITL/R1656/12	MT856818 MT878518*	Faeces	Isernia, Molise	April 12
SVDV	ITL/R1657/12	MT856819 MT878519*	Faeces	Avellino, Campania	April 12
SVDV	ITL/R1658/13	MT856820 MT878520*	Faeces	Reggio Calabria, Calabria	April 13
SVDV	ITL/R1660/14	MT856821 MT878521*	Faeces	Potenza, Basilicata	April 14
SVDV	ITL/R1661/14	MT856822 MT878522*	Faeces	Salerno, Campania	October 14
SVDV	ITL/R1662/14	MT856823 MT878523*	Faeces	Catanzaro, Calabria	November 14
SVDV	Por 1/03	DQ250543	/	/	December 03
SVDV	UKG 27/72	X54521.1	Cc	Staffordshire UK	December 72
PTV	PS-34	/	Cc	Verona, Veneto	/
PTV	O3b	/	Cc	Verona, Veneto	/
PTV	O2b	/	Cc	Verona, Veneto	/
PTV	PS-36	/	Cc	Verona, Veneto	/
PTV	F26	/	Cc	IAH, Pirbright, UK	/
PTV	PS-37	/	Cc	Verona, Veneto	/
PTV	WR2	/	Cc	Verona, Veneto	/
PEV	PS-27	/	Cc	Verona, Veneto	/
PEV	UKG/410/73	/	Cc	IAH, Pirbright, UK	/
PEV	UKG/LP54/75	/	Cc	IAH, Pirbright, UK	/

Footnote: GenBank numbers are referred to the sequenced portions of the 3D codifying gene and of the 5′UTR region, the latter are superscripted with (*); for the reference strain UKG 27/72 the GenBank number refers to the complete genome.
